# 12/15-Lipoxygenase Regulates IL-33-Induced Eosinophilic Airway Inflammation in Mice

**DOI:** 10.3389/fimmu.2021.687192

**Published:** 2021-05-19

**Authors:** Jun Miyata, Yoshiyuki Yokokura, Kazuyo Moro, Hiroyuki Arai, Koichi Fukunaga, Makoto Arita

**Affiliations:** ^1^Laboratory of Metabolomics, RIKEN Center for Integrative Medical Sciences, Yokohama, Japan; ^2^Division of Pulmonary Medicine, Department of Medicine, Keio University School of Medicine, Tokyo, Japan; ^3^Division of Infectious Diseases and Respiratory Medicine, Department of Internal Medicine, National Defense Medical College, Tokorozawa, Japan; ^4^Graduate School of Pharmaceutical Sciences, The University of Tokyo, Tokyo, Japan; ^5^Laboratory for Innate Immune Systems, RIKEN Center for Integrative Medical Sciences (IMS), Yokohama, Japan; ^6^Laboratory for Innate Immune Systems, Department of Microbiology and Immunology, Graduate School of Medicine, Osaka University, Osaka, Japan; ^7^Laboratory for Innate Immune Systems, Immunology Frontier Research Center, Osaka University, Osaka, Japan; ^8^Division of Physiological Chemistry and Metabolism, Keio University Faculty of Pharmacy, Tokyo, Japan; ^9^Cellular and Molecular Epigenetics Laboratory, Graduate School of Medical Life Science, Yokohama City University, Yokohama, Japan

**Keywords:** 12/15-lipoxygenase, IL-33, docosahexaenoic acid, 14(S)-HDoHE, group 2 innate lymphoid cell, lipidomics, maresin, specialized proresolving mediator

## Abstract

Dysregulated fatty acid metabolism is clinically associated with eosinophilic allergic diseases, including severe asthma and chronic rhinosinusitis. This study aimed to demonstrate the role of 12/15-lipoxygenase (12/15-LOX) in interleukin (IL)-33-induced eosinophilic airway inflammation; to this end, we used 12/15-LOX-deficient mice, which displayed augmented IL-33-induced lung inflammation, characterized by an increased number of infiltrated eosinophils and group 2 innate lymphoid cells (ILC2s) in the airway. Liquid chromatography-tandem mass spectrometry (LC-MS/MS)-based lipidomics revealed that the levels of a series of 12/15-LOX-derived metabolites were significantly decreased, and application of 14(S)-hydroxy docosahexaenoic acid (HDoHE), a major 12/15-LOX-derived product, suppressed IL-33-mediated eosinophilic inflammation in 12/15-LOX-deficient mice. Using bioactive lipid screening, we found that 14(S)-HDoHE and 10(S),17(S)-diHDoHE markedly attenuated ILC2 proliferation and cytokine production at micromolar concentration *in vitro*. In addition, maresin 1 (MaR1) and resolvin D1 (RvD1), 12/15-LOX-derived specialized proresolving mediators (SPMs), inhibited cytokine production of ILC2s at nanomolar concentration. These findings demonstrate the protective role of endogenous 12/15-LOX-derived lipid mediators in controlling ILC2-mediated eosinophilic airway inflammation and related diseases. Thus, 12/15-LOX-derived lipid mediators may represent a potential therapeutic strategy for ameliorating airway inflammation-associated conditions.

## Introduction

Asthma is a common disease affecting more than 300 million people worldwide, and the number of patients with asthma is rapidly increasing (1, 2). Genetic and environmental factors induce diverse immune responses that are classified into atopic and non-atopic phenotypes, mainly characterized by eosinophilic airway inflammation. Severe asthma is characterized by resistance to standardized treatments, including corticosteroids, and frequent exacerbation, which could worsen the quality of life in these patients. However, the exact mechanism underlying severe asthma has not been fully elucidated. Thus, it is necessary to elucidate its pathophysiological process.

Dysregulated metabolism of polyunsaturated fatty acids [arachidonic acid (AA), eicosapentaenoic acid (EPA), and docosahexaenoic acid (DHA)] is observed in allergic diseases, including severe asthma and its related diseases, such as aspirin-exacerbated respiratory disease and eosinophilic chronic rhinosinusitis (3, 4). This abnormality is partly characterized by impaired synthesis of specialized proresolving mediators (SPMs; lipoxins (LXs; LXA_4_ and LXB_4_), protectins (PD1 and PDX), resolvin D series (RvDs; RvD1-6), and maresins (MaRs; MaR1-2), which promote the resolution of inflammation. SPMs inhibit the migration of polymorphonuclear cells to inflammatory sites and enhance the phagocytic activity of apoptotic cells *via* macrophages (5, 6). These mediators are mainly biosynthesized *via* 15-lipoxygenase (LOX) in humans and 12/15-LOX, an ortholog of 15-LOX in mice, that are highly expressed in eosinophils and specific types of macrophages (7–10). Previously, we observed downregulated biosynthesis of 15-LOX-derived SPMs in eosinophils isolated from patients with severe asthma and eosinophilic chronic rhinosinusitis (7, 11–13). Systemic administration of SPMs suppressed pulmonary eosinophilic inflammation in murine models of asthma (14–18). These findings suggest the regulatory roles of 15-LOX and 12/15-LOX in eosinophilic inflammation in the lungs, although the causal relationships between these enzymes and severe allergic diseases remain unclear.

Interleukin (IL)-33 is an IL-1 family cytokine with potent inflammatory properties (19). The gene encoding ST2, an IL-33 receptor, is closely related to asthma susceptibility (20, 21). Group 2 innate lymphoid cells (ILC2s) are potent producers of type 2 cytokines, including IL-5 and IL-13, in response to IL-33 (22–24). Other cell types, including eosinophils, basophils, mast cells, and dendritic cells also respond to IL-33, showing proinflammatory reactions (25–35). IL-33 is highly expressed in airway mucosa or nasal polyps isolated from patients with asthma, and its expression level is well correlated with disease severity (36–39). Therefore, IL-33 is considered a pivotal regulator and potential therapeutic target in respiratory diseases with type-2 airway inflammation, including severe asthma.

In the present study, we investigated the regulatory roles of 12/15-LOX-derived lipid mediators in IL-33-induced eosinophilic airway inflammation in mice. Lipidomic analysis of inflamed lung tissue and *in vitro* lipid screening analysis using ILC2s were performed to demonstrate the roles of 12/15-LOX.

## Materials and Methods

### Mouse Experiments

Specific pathogen- and virus-antibody-free, 6-8-week-old, male C57BL/6J (C57BL/6) mice, weighing 25-30 g, were purchased from Charles River Laboratories, Japan. 12/15-LOX-deficient mice were obtained from the Jackson Laboratory (002778, Bar Harbor, ME, USA). All animals were housed at the facility in bubble barrier units (bioBubble, Fort Collins, Colo., USA) under positive pressure. The experimental protocol was reviewed and approved by the Laboratory Animal Care and Use Committee of Keio University of Medicine, the Animal Committee of the University of Tokyo, and the Animal Care and Use Committee of the RIKEN.

### Administration of Reagents *In Vivo*

We administered 40 μL of PBS or IL-33 (R&D, 500 ng per mouse) *via* intranasal administration under anesthesia with intraperitoneal administration of ketamine (100 mg/kg) and xylazine (10 mg/kg). In some experiments, we simultaneously administered 5 μg/day of 14(S)-HDoHE (Cayman Chemical, Ann Arbor, MI, USA) *via* intraperitoneal injection.

### Establishment of IL-33-Induced Airway Inflammation

We administered IL-33 (R&D Systems, Minneapolis, MN, 500 ng per mouse) for 3 consecutive days and analyzed them 1 or 4 days after the last challenge as previously described (40) with technical modification. Bronchoalveolar lavage fluid (BALF) was collected for cell counts and flow cytometric analysis, and lung tissue for the measurement of mRNA expression and histopathologic analysis. Formalin-fixed paraffin-embedded lung slides were stained with hematoxylin and eosin (HE) or periodic acid Schiff (PAS).

### Collection of BALF

The mice were sacrificed by an overdose of intravenous pentobarbital at the indicated times after the last challenge. The trachea was cannulated, and the lungs were lavaged by washing twice with 0.7 mL of ice-cold PBS with EDTA (0.6 mM). The total number of cells in BALF was counted using a hemocytometer, and a differential cell count of 200 cells was determined on Diff-Quik-stained cytospin slides (Baxter Scientific Products, McGraw Park, Ill., USA) prepared with Auto Smear CF12D (Sakura Finetek, Tokyo, Japan). Flow cytometric analysis was performed for cell counts of specific types of lymphocytes (ILC2s and Th2 cells).

### Targeted Liquid Chromatography Tandem Mass Spectrometry (LC-MS/MS)-Based Lipidomics

LC-MS/MS-based mediator lipidomics was performed as previously described (41). Lung tissues were homogenized in ice-cold methanol and kept in -20°C overnight. The methanolic extract was then diluted with water, acidified with HCl to a pH of 3.5, and applied to Sep-Pak C18 cartridges (Waters) for solid phase extraction. Deuterated internal standards (1 ng of leukotriene (LT) B_4_-d4, LTD_4_-d5, prostaglandin (PG) E_2_-d4, and 15-HETE-d8 (Cayman Chemical, Ann Arbor, MI, USA) were added to the supernatants prior to extraction. For LC-MS/MS analysis, a triple quadrupole linear ion trap mass spectrometer (QTRAP 5500; AB Sciex, Foster City, CA) equipped with a 1.7-μm, 1.0 × 150 mm Acquity UPLC™ BEH C18 column (Waters Corp., Milford, MA) was used. MS/MS analyses were conducted in negative ion mode, and the eicosanoids and docosanoids were identified and quantified by multiple reaction monitoring. Calibration curves were obtained over a range of 1–1,000 pg. The LC retention times for each compound were determined using the corresponding synthetic standards. PD1 and PDX, stereoisomers, were not separable under this LC-MS/MS setting.

### Functional Assays of ILC2s *In Vitro*

Mouse ILC2s were isolated from mesentery using a previously reported method (42). Purified ILC2s were cultured in 96-well round-bottom plates in 200 μL RPMI-1640 media containing 10% FCS, HEPES buffer, non-essential amino acids, penicillin, streptomycin, and 2-mercaptoethanol in the presence of IL-2 (10 ng/mL) at 37°C. To comprehensively evaluate the effect of lipid metabolites, cultured ILC2s were seeded at a density of 10,000 cells per well into 96-well round-bottom plates in the absence of IL-2, and IL-33 was added to the culture medium at a final concentration of 10 ng/mL after pretreatment for 30 min with lipid metabolites (10^-11^ M – 10^-5^ M). One hundred microliters of supernatant were collected for the cytokine assay, and ILC2s were counted by flow cytometry on day 4. For apoptosis analysis, ILC2s were stained with Annexin V and propidium iodide (PI) according to the manufacturer’s protocols (Apoptosis Detection Kit, BD Pharmingen), and then analyzed by flow cytometry on day 1 and day 4. All data were analyzed using FlowJo software (TreeStar, Ashland, OR, USA).

### Statistical Analysis

Data are presented as the mean ± SEM. Dose-response relationships of lipid metabolites on ILC2 activities were analyzed with repeated measures of analysis of variance, followed by the Bonferroni/Dunn procedure as a *post hoc* test. Data were analyzed using GraphPad Prism version 4.0c (GraphPad Software, San Diego, CA). Statistical significance was set at P < 0.05.

## Results

### 12/15-LOX Deficiency Augmented IL-33-Induced Airway Eosinophilic Inflammation

**Figure 1:**
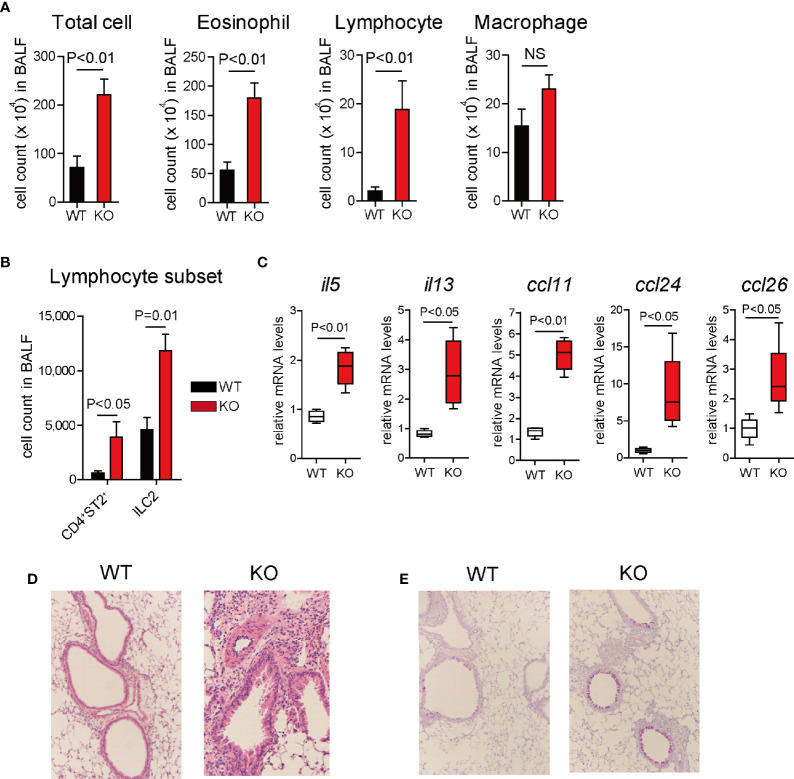
12/15-lipoxygenase deficiency augments IL-33-induced airway eosinophilic inflammation. Airway inflammation was induced by administration of IL-33 (500 ng per mouse, intranasal), for 3 consecutive days, in C57BL/6 and 12/15-lipoxygenase deficient mice. Analysis was performed 4 days after the final administration of IL-33. **(A)** Number of total cells, eosinophils, lymphocytes, and macrophages in BALF. **(B)** Number of ILC2 and Th2 cells in BALF by flow cytometric analysis. **(C)** Relative mRNA expression of cytokines (*il5*, *il13*, *ccl11*, *ccl24*, and *ccl26*) was determined by RT-PCR and quantitative real-time PCR analysis. Airway histology was assessed by **(D)** hematoxylin and eosin and **(E)** periodic acid–Schiff (PAS)-Alcan blue staining. The data are representative of three independent experiments. Mean ± SEM, n=4 for each group.

To determine whether 12/15-LOX affects the disease onset and/or progression of airway eosinophilic inflammation, we used mice deficient in the gene encoding 12/15-LOX (*alox15*) in a murine model of IL-33-induced innate airway eosinophilic inflammation. 12/15-LOX-deficient (12/15-LOX^-/-^) mice developed more severe airway inflammation associated with an increased number of eosinophils and lymphocytes in BALF compared to controls (**Figure 1A**). Lymphocyte subset analysis revealed a significant increase in the number of ILC2s and Th2 cells (CD4^+^ST2^+^ cells) in BALF from IL-33-challenged 12/15-LOX^-/-^ mice compared to wild-type mice (**Figure 1B**). Importantly, the number of ILC2s was greater than that of Th2 cells (**Figure 1B**). Expression of mRNA levels of type 2 cytokines (*il5*, *il13*) and chemokines for eosinophils (*ccl11*, *ccl24*, *and ccl26*), which are critical inducers of eosinophilic inflammation, in the lungs was significantly higher in IL-33-treated 12/15-LOX^-/-^ mice than in wild-type mice (**Figure 1C**). Histological analysis demonstrated prominent accumulation of inflammatory cells around the bronchus and increased mucus production in the lung tissue stained with HE and PAS, respectively (**Figures 1D, E**). In addition, the absence of 12/15-LOX had no impact on the number of cells and lymphocyte subset including Th2 cells and ILC2s in BALF at the steady state (Supplementary **Figures 1A, B**).

### Lipidomic Profile of Lung Tissue During IL-33-Induced Airway Eosinophilic Inflammation

**Figure 2:**
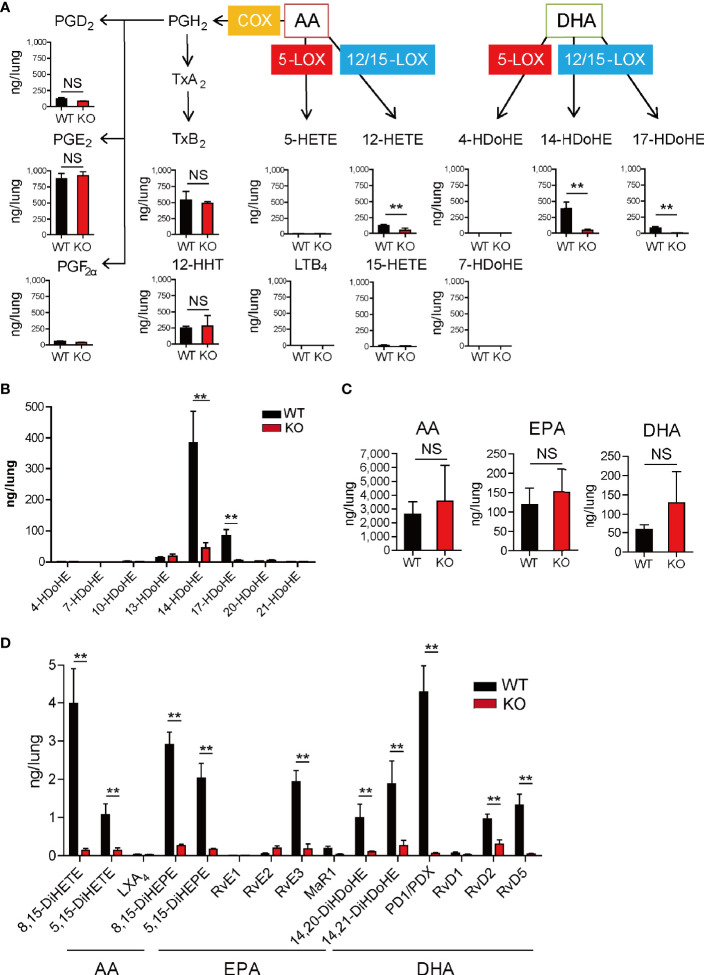
Lipidomic profiles of lungs during IL-33-induced airway eosinophilic inflammation. Airway inflammation was induced by administration of IL-33 (500 ng per mouse, intranasal), for 3 consecutive days, in C57BL/6 and 12/15-lipoxygenase (LOX)-deficient mice. Analysis was performed 1 day after the final administration of IL-33. **(A)** Lipidomic analysis showed quantitative alterations of arachidonic acid (AA) and docosahexaenoic acid (DHA)-derived metabolites *via* cyclooxygenase (COX), 5-LOX, and 12/15-LOX, including prostaglandins (PG), thromboxanes (Tx), 12- hydroxyheptadecatrienoic acid (HHT), hydroxyeicosatetraenoic acid (HETE), leukotriene B_4_ (LTB_4_), and hydroxy docosahexaenoic acid (HDoHE). **(B)** Comparative analysis of DHA-derived monohydroxy metabolites in wild-type or 12/15-LOX deficient mice. **(C)** Comparative analysis of the amounts of polyunsaturated fatty acids [AA, DHA, and eicosapentaenoic acid (EPA)] in wild-type or 12/15-LOX deficient mice. **(D)** Comparative analysis of 12/15-LOX-derived dihydroxy- or trihydroxy-lipid metabolites, including lipoxin (LX), Rv, MR, and protectin (PD). Mean ± SEM, n=3 for each group. **P < 0.01 (Student’s t-test). NS, not significant.

To investigate the lipid mediator profiles in the lungs during IL-33-induced eosinophilic pulmonary inflammation, LC-MS/MS-based mediator lipidomics was performed. The amounts of lipid mediators on days 1 and 4 following the last challenge are summarized in **Supplementary Table 1**. COX-derived products such as PGE_2_, PGD_2_, and PGF_2α_, thromboxane B_2_, and 12-HHT were abundantly produced in the inflamed lung tissue on day 1, and these amounts were almost the same between wild-type and 12/15-LOX^-/-^ mice (**Figure 2A**). Among the LOX-derived products, 14-HDoHE, 17-HDoHE, and 12-hydroxyeicosatetraenoic acid (HETE) were generated at substantial levels on day 1. Additionally, their biosynthesis was dependent on 12/15-LOX (**Figure 2A**). Among the monohydroxylated forms of DHA (4-, 7-, 10-, 13-, 14-, 17-, 20-, and 21-HDoHE), the amount of 14-HDoHE was the highest on day 1 (**Figure 2B**). The amounts of fatty acids, precursors of lipid mediators (AA, EPA, and DHA), in the lungs did not differ between wild-type and 12/15-LOX^-/-^ mice on day 1 (**Figure 2C**). Among the downstream lipid mediators (AA-, EPA-, and DHA-derived dihydroxy- or trihydroxy-fatty acids), PD1/PDX, RvD2, RvD5, and RvE3 were also present in a 12/15-LOX-dependent manner on day 1 (**Figure 2D**). PD1/PDX was relatively abundant compared to other mediators.

### 14(S)-HDoHE and 10(S),17(S)-diHDoHE Suppress ILC2 Cell Activation *In Vitro*

**Figure 3:**
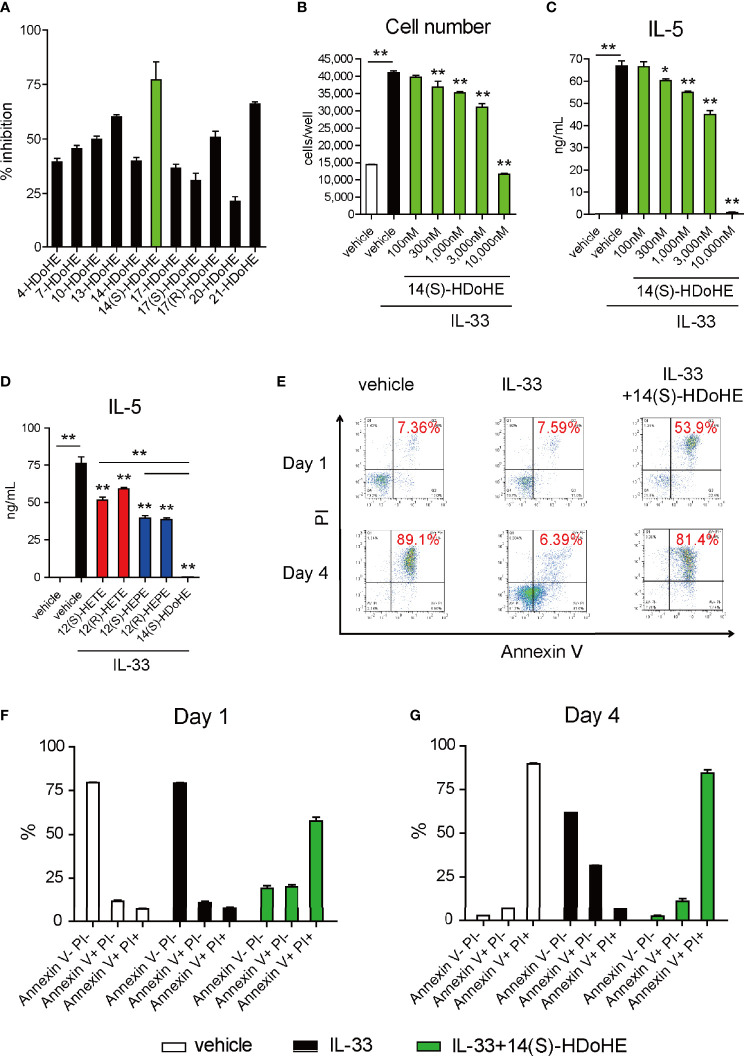
14(S)-HDoHE inhibit proliferation and cytokine production of ILC2s, with pro-apoptotic effects. ILC2 cells (10,000 cells per well) were cultured with IL-33 (10 ng/mL) for 1 or 4 days in the presence or absence of **(A)** DHA-derived monohydroxy-lipid metabolites (HDoHE, hydroxy docosahexaenoic acid: 10^-5^ M) or **(B–G)** in the presence or absence of 14(S)-HDoHE (3 × 10^-7^ – 10^-5^ M: **(B, C)**; or 10^-5^ M: **(D–G)**, **(D)** 12-hydroxyeicosatetraenoic acid (HETE) and 12-hydroxyeicosapentaenoic acid (HEPE). The total cell count **(A, B)** and concentrations of IL-5 **(C, D)** in the culture supernatant were measured. **(E–G)** Flow cytometric analysis of Annexin V- and PI-stained ILC2 cells cultured with or without IL-33 (10 ng/mL) in the presence or absence of 14(S)-HDoHE (10^-5^ M) for 1 or 4 days. The data are representative of three independent experiments. Mean ± SEM, n=3-8 for each group. *P < 0.05, **P < 0.01 (Student’s t-test).

**Figure 4:**
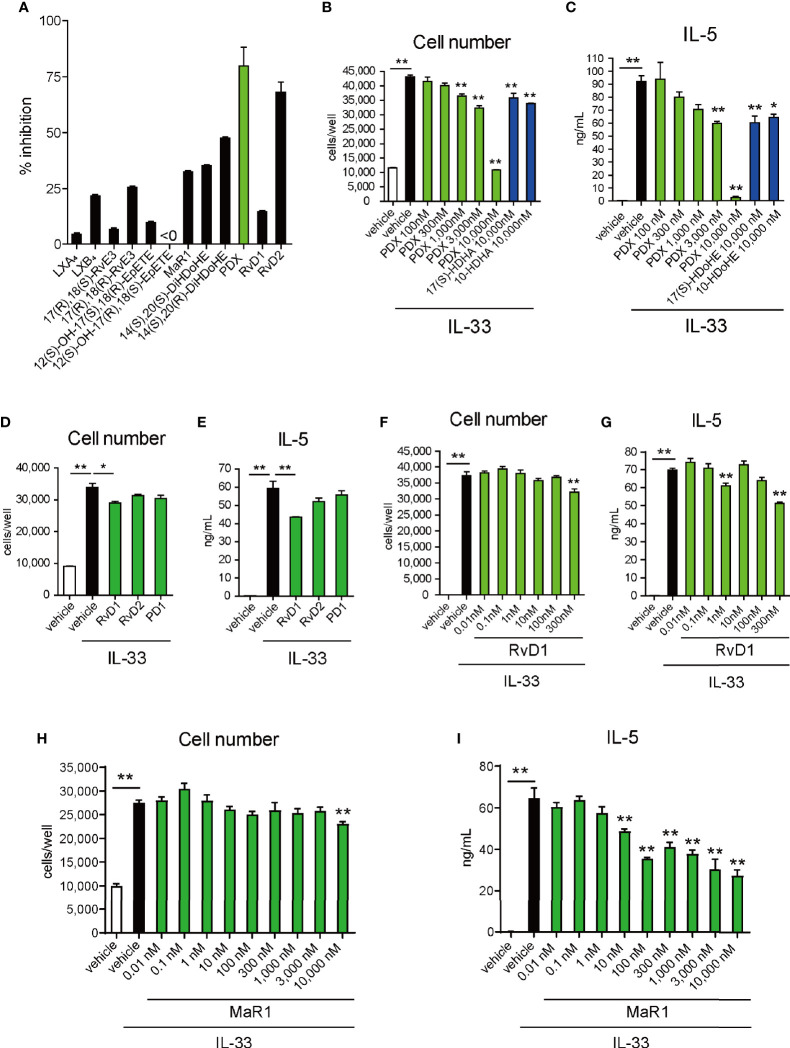
Effects of SPMs on proliferation and cytokine production of ILC2s. **(A)** ILC2 cells (10,000 cells per well) were cultured with IL-33 (10 ng/mL) for 4 days in the presence or absence of **(A)** dihydroxy- or trihydroxy-lipid metabolites (LX, lipoxin; Rv, resolvin; MR, maresin; PD, protectin D: 10^-5^ M) or in the presence or absence of **(B, C)** 10(S),17(S)-diHDoHE (PDX, 3 × 10^-7^ – 10^-5^ M), **(D, E)** 17(S)-HDoHE-derived SPMs (RvD1, resolvin D1; RvD2, resolvin D2; PD1, protectin D1: 3 x 10^-7^ nM), **(F, G)** RvD1 (1 × 10^-11^ – 3 x 10^-7^ M: **(D, E)**, or **(H, I)** MaR1 (MaR1; maresin 1: 10^-11^ – 10^-5^ M). The total cell count **(A, B, D, F, H)** and concentrations of IL-5 **(C, E, G, I)** in the culture supernatant were measured. Data are shown as Mean ± SEM, n = 3 for each group. *P < 0.05, **P < 0.01 (Student’s *t*-test).

To further understand the molecular mechanism of 12/15-LOX in the regulation of innate eosinophilic pulmonary inflammation, we performed lipid screening using AA-, EPA-, and DHA-derived metabolites. Their suppressive effects on the proliferation of ILC2 cells *in vitro* in response to IL-33 were investigated by flow cytometry. The lipid screening assay demonstrated that 14(S)-HDoHE was a potent suppressor of cellular proliferation of ILC2s among DHA-derived monohydroxy metabolites (4-, 7-, 10-, 13-, 14-, 17-, 20-, and 21-HDoHE) (**Figure 3A**). In addition, among DHA-derived SPMs, including RvDs, PDs, MaRs, and other AA- or EPA-derived dihydroxy or trihydroxy metabolites, 10(S),17(S)-diHDoHE (PDX) most potently suppressed the proliferation of ILC2 cells (**Figure 4A**).

14(S)-HDoHE suppressed ILC2 cell proliferation in a concentration-dependent manner (**Figure 3B**). In addition, cytokine release (IL-5) from ILC2 cells was suppressed by 14(S)-HDoHE in a concentration-dependent manner (**Figure 3C**). The inhibitory effect of 14(S)-HDoHE on IL-5 production was more potent than other related structural isomers such as 12(S)-HETE, 12(R)-HETE, 12(S)-HEPE, and 12(R)-HEPE (**Figure 3D**). Flow cytometric analysis of Annexin V- and PI-stained ILC2 cells upon stimulation with IL-33 revealed the pro-apoptotic effects of 14(S)-HDoHE on ILC2s after stimulation with IL-33 for 1 or 4 days (**Figures 3E–G**). PDX similarly inhibited ILC2 proliferation and IL-5 production in a concentration-dependent manner. PDX displayed more potent suppressive effect than its related metabolites including 17(S)-HDoHE and 10(S/R)-HDoHE (**Figures 4B, C**). The suppressive effects of PD1, RvD1, and RvD2, were also evaluated at nanomolar concentrations. Unlike the others, RvD1 slightly inhibited the proliferation and IL-5 production of ILC2s (**Figures 4D–G**). In addition, the suppressive effects of MaR1 were evaluated at nanomolar to micromolar concentrations. MaR1 inhibited IL-5 production, not cell proliferation, of ILC2s at nanomolar concentrations (**Figures 4H, I**). These results suggest that the DHA-derived 12/15-LOX metabolites collectively regulates ILC2 functions to control innate type-2 airway inflammation.

### 14(S)-HDoHE Suppressed IL-33-Induced Airway Eosinophilic Inflammation *In Vivo*

**Figure 5:**
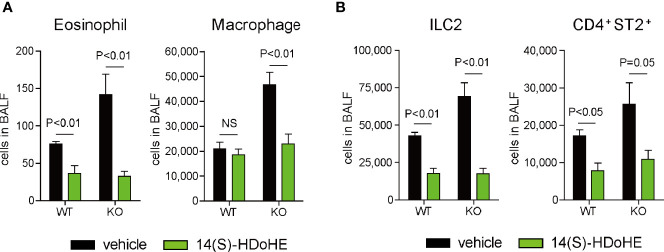
14(S)-HDoHE suppresses IL-33-induced airway eosinophilic inflammation. Airway inflammation was induced by administration of IL-33 (500 ng per mouse, intranasal), for 3 consecutive days, in C57BL/6 and 12/15-lipoxygenase deficient mice. 14(S)-hydroxy docosahexaenoic acid (HDoHE) was administered *via* intraperitoneal injection prior to IL-33 administration. Flow cytometric analysis was performed 4 days after the final administration of IL-33. **(A)** Number of eosinophils and macrophages in the BALF. **(B)** Number of lymphocyte subsets, including ILC2 and Th2 cells, in the BALF. Mean ± SEM, n=4 for each group. NS, not significant.

Next, we determined the potential preventive effect of 14(S)-HDoHE, a major product of 12/15-LOX in the lung during inflammation, *in vivo* on IL-33-induced airway eosinophilic inflammation. Intraperitoneal administration of 14(S)-HDoHE reduced the number of eosinophils and macrophages in BALF compared with that in vehicle-treated mice in 12/15-LOX^-/-^ mice (**Figure 5A**). 14(S)-HDoHE administration also decreased the number of ILC2 and CD4^+^ST2^+^ Th2 cells present in BALF in 12/15-LOX^-/-^ mice (**Figure 5B**). Also, 14(S)-HDoHE reduced the number of ILC2 and Th2 cells in BALF of WT mice (**Figure 5B**).

## Discussion

In the present study, we demonstrated that 12/15-LOX, a key enzyme for the biosynthesis of specialized pro-resolving lipid mediators (SPMs), conferred a protective effect on innate pulmonary eosinophilic inflammation *in vivo*. Lipidomic analysis revealed a series of 12/15-LOX-derived mediators present at substantial levels in the inflamed lung. Using bioactive lipid screening, the potent effect of 14(S)-HDoHE and 10(S),17(S)-diHDoHE in suppressing ILC2 function was observed *in vitro*. These findings demonstrate the direct effect of DHA-derived pro-resolving mediators in suppressing ILC2 activation. 14(S)-HDoHE also displayed potent anti-inflammatory effects on IL-33-induced eosinophilic airway inflammation when administered *in vivo*. These results provide a new therapeutic option for 12/15-LOX-derived pro-resolving mediators in ILC2-mediated allergic diseases.

Previous studies have shown the regulatory effects of 14(S)-HDoHE on murine platelets and human alveolar macrophages *in vitro* (43, 44). PD1/PDX also possesses potent anti-inflammatory functions in regulating neutrophil activation *in vitro* (45, 46) and ameliorates pulmonary inflammation and fibrosis *in vivo* (47, 48). Interestingly, we previously reported that peripheral blood eosinophils isolated from patients with severe asthma had a defective biosynthetic capacity of 14(S)-HDoHE, 17(S)-HDoHE, and PD1/PDX (7). Similarly, their biosynthetic capacities have been reported to be impaired in obese mice, and systemic administration of these metabolites ameliorated obesity-induced inflammatory states (49). These findings highlight the therapeutic potential of 14(S)-HDoHE and related SPMs in the regulation of chronic inflammation through DHA metabolism. However, the precise mechanism underlying the 14(S)-HDoHE-mediated effects thorough specific receptors and/or downstream metabolites remains undetermined.

IL-33 induces ILC2 proliferation and activation, which orchestrate innate type-2 inflammation as the dominant IL-5-producing cell *in vitro* and *in vivo* (22, 23, 50–55). IL-5 plays a central role in prolonging eosinophils survival and activating eosinophils to elicit degranulation, superoxide generation, cytokine release, and cysteinyl leukotriene synthesis (12, 56). In this study, 14(S)-HDoHE, 17(S)-HDoHE, and other DHA-derived SPMs, such as RvDs, PDs, and MaRs, differentially exerted regulatory effects on cellular functions of ILC2 cells at micromolar or nanomolar concentrations. In humans, LXA_4_, an AA-derived SPM, decreased IL-13 release from ILC2s stimulated with IL2/IL25/IL-33/PGD_2_ at nanomolar concentrations (57). In addition, MaR1, a DHA-derived SPM, regulated IL-13 release from ILC2 *in vitro* at nanomolar concentration and *in vivo* at ng/mouse administration in a murine model of asthma (58). Also, regulatory T cells synergistically exerted inhibitory effects on ILC2 in the presence of MaR1 (58). Other cell types, including regulatory T cells, may enhance the suppressive effects of SPMs on ILC2s. Additionally, our study suggests that RvD1 and MaR1 can inhibit ILC2 function at nanomolar concentration. Thus, we speculate that these bioactive SPMs collectively, not individually, orchestrate the regulatory circuit for ILC2-mediated eosinophilic inflammation.

The cellular sources of 12/15-LOX-derived mediators during allergic inflammation are of particular interest. 12/15-LOX-expressing eosinophils play pro-resolving functions by enhancing the resolution of neutrophilic inflammation in acute peritonitis (8, 9) and by promoting corneal wound healing in the eye (59). In addition, 12/15-LOX-expressing resident macrophages play an important role in the efferocytosis of apoptotic neutrophils in acute peritonitis (10). Further studies are required to identify the cell types that locally produce 12/15-LOX-derived SPMs to suppress ILC-2-mediated eosinophilic inflammation.

In conclusion, our results demonstrate that 12/15-LOX-derived mediators regulate IL-33-induced eosinophilic airway inflammation. Bioactive lipid screening identified 14(S)-HDoHE and 10(S),17(S)-diHDoHE as potent endogenous suppressors of ILC2 activation. These findings contribute to a better understanding of the cellular and molecular mechanisms underlying the resolution of eosinophilic airway inflammation. Thus, 12/15-LOX-derived lipid mediators and/or 12/15-LOX-mediated lipid metabolism may represent a potential therapeutic strategy for ameliorating airway inflammation-associated conditions.

## Data Availability Statement

The raw data supporting the conclusions of this article will be made available by the authors, without undue reservation.

## Ethics Statement

The animal study was reviewed and approved by the Animal Care and Use Committee of the RIKEN, the Animal Committee of the University of Tokyo, the Laboratory Animal Care and Use Committee of Keio University of Medicine.

## Author Contributions

JM and MA designed the experiments. JM and YY performed most of the experiments and compiled the data. KM provided murine group 2 innate lymphoid cells. MA, KF, and HA supervised the project. JM and MA wrote the manuscript, and all authors provided feedback on the manuscript. All authors contributed to the article and approved the submitted version.

## Funding

This work was supported by the JSPS Grant-in-Aid for Scientific Research on Innovative Areas (15H05897, 15H05898 to MA), the RIKEN Special Postdoctoral Researchers Program (to JM), and the Grant-in-Aid for research of ONO Medical Research Foundation (to JM).

## Conflict of Interest

The authors declare that the research was conducted in the absence of any commercial or financial relationships that could be construed as a potential conflict of interest.

## Abbreviations

Alox15, 12/15-lipoxygenase; AA, arachidonic acid; BALF, bronchial alveolar lavage fluid; DHA, docosahexaenoic acid; EPA, eicosapentaenoic acid; HDoHE, hydroxy docosahexaenoic acid; HE, hematoxylin eosin; HETE, hydroxyeicosatetraenoic acid; IL, interleukin; LC, liquid chromatography; LOX, lipoxygenase; LT, leukotriene; MaRs, maresins; MS/MS, tandem mass spectrometry; PAS, periodic acid Schiff; PD1, protectin D1; PDX, protectin DX; PG, prostaglandin; RT-PCR, reverse transcription-PCR; Rv, resolvin; SPM, specialized proresolving mediator.
