# Supplementary material for: 12/15-Lipoxygenase Regulates IL-33-Induced Eosinophilic Airway Inflammation in Mice
Source: Front Immunol. 2021 May 19;12:687192. doi: 10.3389/fimmu.2021.687192 (PMC8170304; doi:10.3389/fimmu.2021.687192)
Supplement: Supplementary file 1 [file DataSheet_1.docx]

12/15-Lipoxygenase Regulates IL-33-induced Eosinophilic Airway Inflammation in Mice

Jun Miyata^1,2,3^, Yoshiyuki Yokokura^4^, Kazuyo Moro^5,6,7^, Hiroyuki Arai^4^, Koichi Fukunaga^2^, Makoto Arita^1,8,9*^

^1^ Laboratory of Metabolomics, RIKEN Center for Integrative Medical Sciences, 1-7-22 Suehiro-cho, Tsurumi-ku, Yokohama, Kanagawa, Japan

**^2^** Division of Pulmonary Medicine, Department of Medicine, Keio University School of Medicine, 35 Shinanomachi, Shinjuku-ku, Tokyo, Japan

^3^ Division of Infectious Diseases and Respiratory Medicine, Department of Internal Medicine, National Defense Medical College, 3-2 Namiki, Tokorozawa, Saitama, Japan

^4^ Graduate School of Pharmaceutical Sciences, The University of Tokyo, 7-3-1 Hongo, Bunkyo-ku, Tokyo, Japan

^5^ Laboratory for Innate Immune Systems, RIKEN Center for Integrative Medical Sciences (IMS), 1-7-22 Suehiro-cho, Tsurumi-ku, Yokohama, Kanagawa, Japan

^6^ Laboratory for Innate Immune Systems, Department of Microbiology and Immunology, Graduate School of Medicine, Osaka University, 2-2 Yamadaoka Suita-shi, Osaka, Japan

^7^ Laboratory for Innate Immune Systems, IFReC, Osaka University, 3-1 Yamadaoka Suita-shi, Osaka, Japan

^8^ Division of Physiological Chemistry and Metabolism, Keio University Faculty of Pharmacy, 1-5-30 Shibakoen, Minato-ku, Tokyo, Japan

^9^ Cellular and Molecular Epigenetics Laboratory, Graduate School of Medical Life Science, Yokohama City University, 1-7-29 Suehiro-cho, Tsurumi-ku, Yokohama, Kanagawa, Japan

*** Correspondence:**Makoto Arita
makoto.arita@riken.jp

Supplementary material

# Figure Legend

**Supplementary Figure 1. Comparison of cells in BALF between C57BL/6 and 12/15-lipoxygenase deficient mice at steady state.** Analysis of BALF from C57BL/6 and 12/15-lipoxygenase deficient mice was performed at steady state. (A) Total number of cells, eosinophils, lymphocytes, and macrophages in BALF. (B) Number of ILC2 and Th2 cells in BALF determined using flow cytometric analysis. Data are shown as Mean ± SEM, n = 3 for each group.

**Supplementary Table 1. The lipid mediator profiles in the lungs during inflammation**

| Mediators | Day 1 | | | | Day 4 | | | |
| --- | --- | --- | --- | --- | --- | --- | --- | --- |
|  | WT (n = 3) | | Alox15KO (n = 3) | | WT (n = 3) | | Alox15KO (n = 3) | |
|  | Mean | SEM | Mean | SEM | Mean | SEM | Mean | SEM |
| Arachidonic Acid |  |  |  |  |  |  |  |  |
| PGD_2_ | 127,018.6 | 11,012.5 | 83,426.1 | 3,529.0 | 3,406.1 | 696.0 | 769.2 | 535.8 |
| 15-deoxy PGJ_2_ | 28.9 | 5.4 | 216.9 | 115.2 | 84.8 | 10.5 | 95.7 | 25.6 |
| PGE_2_ | 874,883.0 | 81,345.3 | 922,359.7 | 60,631.5 | 42,268.7 | 5,237.1 | 9,199.0 | 7,567.7 |
| PGF_2a_ | 56,609.6 | 4,714.4 | 40,850.7 | 6,578.8 | 1,314.5 | 243.8 | 388.4 | 291.2 |
| TxB_2_ | 537,447.7 | 135,151.5 | 489,475.8 | 27,283.6 | 17,117.7 | 3,731.9 | 4,602.0 | 371.9 |
| 12-HHT | 248,626.6 | 26,447.6 | 277,713.0 | 166,079.8 | 8,267.6 | 2,575.9 | 2,920.2 | 520.9 |
| LTB_4_ | 26.0 | 9.8 | 38.8 | 21.3 | 7.2 | 3.2 | 0.0 | 0.0 |
| 5-HETE | 5,014.2 | 965.8 | 3,359.9 | 1,084.4 | 581.2 | 85.0 | 282.8 | 85.5 |
| 5-oxo ETE | 358.2 | 91.4 | 353.4 | 118.0 | 74.4 | 28.6 | 86.0 | 30.4 |
| 12-HETE | 128,258.9 | 22,970.4 | 49,378.3 | 36,297.6 | 18,999.4 | 5,316.6 | 145.5 | 83.2 |
| 15-HETE | 19,458.7 | 3,746.9 | 13,648.9 | 4,335.5 | 5,314.9 | 1,282.1 | 155.6 | 45.2 |
| LXA_4_ | 13,454.1 | 4,058.9 | 11,084.9 | 604.6 | 2,146.8 | 806.5 | 756.4 | 286.0 |
| Docosahexaenoic acid |  |  |  |  |  |  |  |  |
| 4-HDoHE | 890.9 | 134.2 | 1,116.9 | 558.3 | 163.7 | 8.7 | 373.5 | 191.4 |
| 7-HDoHE | 248.7 | 114.3 | 158.9 | 13.2 | 38.8 | 6.1 | 41.1 | 8.1 |
| 13-HDoHE | 13,650.2 | 2,726.0 | 18,859.3 | 6,369.1 | 921.3 | 98.9 | 130.0 | 44.0 |
| 14-HDoHE | 384,564.9 | 101,023.9 | 45,553.1 | 16,027.7 | 60,131.2 | 4,319.0 | 229.6 | 73.9 |
| 17-HDoHE | 83,997.9 | 19,771.6 | 5,477.2 | 1,443.7 | 36,893.0 | 6,266.8 | 258.2 | 92.9 |
| 20-HDoHE | 2,681.3 | 296.1 | 5,883.2 | 1,302.2 | 206.0 | 36.6 | 277.9 | 34.9 |
| 21-HDoHE | 822.8 | 265.6 | 972.1 | 386.1 | 226.6 | 29.2 | 377.1 | 61.1 |
| 4,14-DiHDoHE | 1,661.7 | 451.3 | 148.0 | 34.0 | 517.5 | 25.7 | 100.5 | 5.4 |
| Maresin | 194.8 | 59.1 | 41.5 | 10.5 | 122.3 | 84.6 | 83.4 | 11.2 |
| 14,20-DiHDoHE | 994.5 | 352.5 | 103.2 | 20.4 | 317.8 | 134.8 | 121.2 | 60.6 |
| 14,21-DiHDoHE | 1,878.4 | 602.6 | 263.1 | 143.6 | 1,369.9 | 424.9 | 263.7 | 138.5 |
| PD1 | 4,288.8 | 691.0 | 58.9 | 31.0 | 2,033.8 | 104.8 | 44.1 | 22.5 |
| RvD1 | 74.7 | 19.1 | 32.7 | 16.0 | 14.0 | 3.4 | 40.2 | 17.2 |
| RvD2 | 966.6 | 124.8 | 305.0 | 108.7 | 286.9 | 52.5 | 46.8 | 13.1 |
| RvD5 | 1,320.7 | 293.7 | 42.9 | 17.1 | 207.0 | 21.7 | 42.2 | 10.9 |
| Data are expressed in pg/mouse. | | | | | | | | |
