# Supplementary figures and images for: 12/15-Lipoxygenase Regulates IL-33-Induced Eosinophilic Airway Inflammation in Mice
Source: Front Immunol. 2021 May 19;12:687192. doi: 10.3389/fimmu.2021.687192 (PMC8170304; doi:10.3389/fimmu.2021.687192)

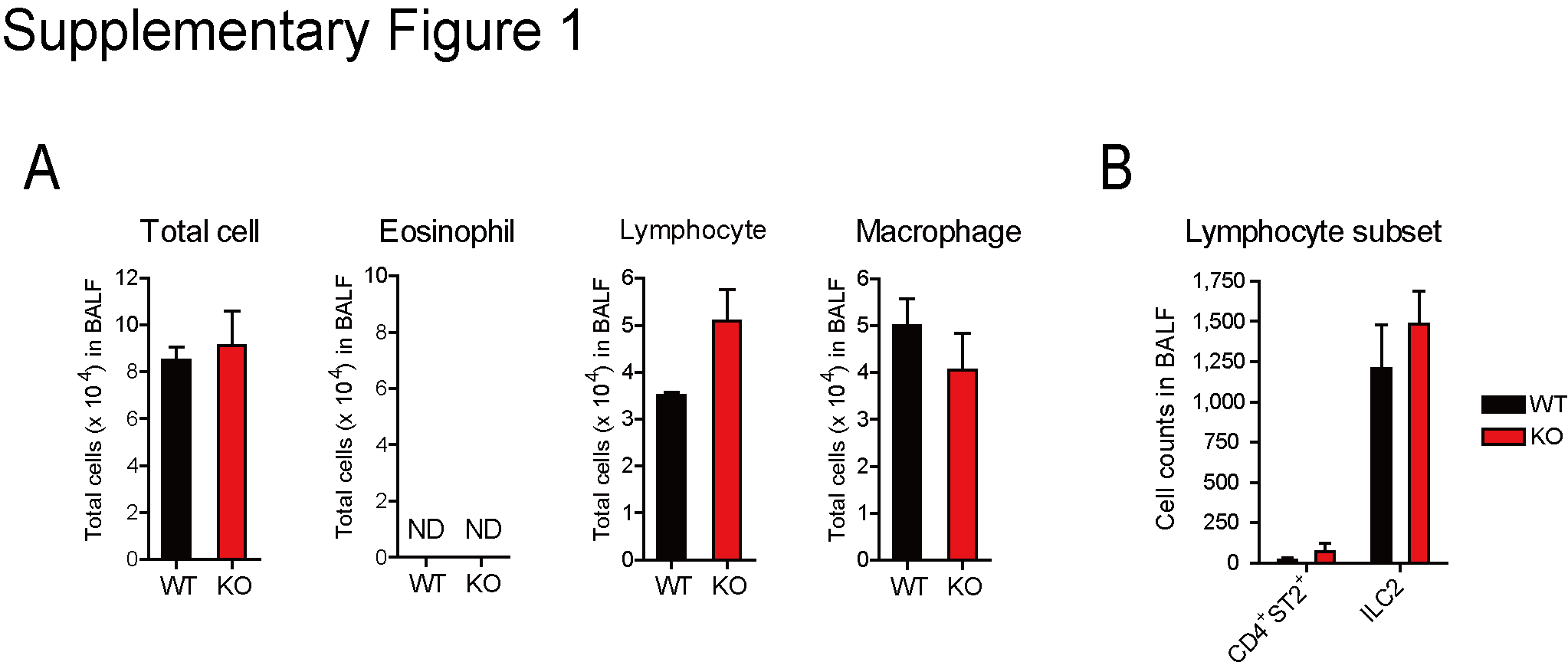

Supplement: Supplementary file 2 [file Image_1.tif]
